# How understanding and strengthening brain networks can contribute to elementary education

**DOI:** 10.3389/fpubh.2023.1199571

**Published:** 2023-06-15

**Authors:** Michael I. Posner, Mary K. Rothbart

**Affiliations:** Department of Psychology, University of Oregon, Eugene, OR, United States

**Keywords:** attention, memory, elementary education, number, reading, mindset

## Abstract

Imaging the human brain during the last 35 years offers potential for improving education. What is needed is knowledge on the part of educators of all types of how this potential can be realized in practical terms. This paper briefly reviews the current level of understanding of brain networks that underlie aspects of elementary education and its preparation for later learning. This includes the acquisition of reading, writing and number processing, improving attention and increasing the motivation to learn. This knowledge can enhance assessment devices, improve child behavior and motivation and lead to immediate and lasting improvements in educational systems.

## 1. Introduction

In the late 20th century, it became possible to examine the living human brain during the performance of cognitive tasks, including those normally taught in school [see (1) for a review]. The main method of doing this is to place the person in a magnetic resonance imager. The signal detected in functional magnetic resonance imaging (fMRI) reflects changes driven by localized brain blood flow and blood oxygenation, which are coupled to the level of neuronal activity. This allows construction of an image of the brain marked with the areas of increased neural activity.

From the earliest studies of brain imaging it was clear that even very simple tasks, like retrieving the use of a “hammer”, activated neurons in several widely separated cortical and subcortical brain areas related to different aspects of language. During the last 35 years new methods of imaging the brain have also been developed. One of these, Diffusion Tensor Imaging (DTI) allows connections between active areas of the brain to be imaged.

Many of the brain networks imaged in adults performing tasks like reading are also active when the person is not performing a task, but is in a resting state (2). The ability to image networks in the resting brain allows them to be studied even in infancy, when the baby is not able to perform a task. Language networks have been imaged from birth using this resting state method (3). Thus, during the early years of this century a tool kit of methods to study brain networks related to cognitive tasks and the resting state has been developed.

While books (4), scholarly articles and even podcasts have attempted to inform teachers and others involved in education about the relevance of brain developments, recent articles have noted widespread failure of current methods to reflect the relevance of brain imaging findings to education (5, 6). The implication of brain research for teaching in elementary schools has either not been understood or failed to be applied for other reasons.

The goal of this paper is to make clear findings in the human brain that could influence elementary school education, improving learning by students and increasing their understanding of why the effort required of them is worthwhile. We attempt to outline in a non-technical way the specific brain networks related to instruction in reading and number that are critical for elementary school subjects. In addition we discuss attention and motivation as important tools for learning. Finally, we discuss concrete steps that teachers may consider taking to assist brain changes that occur while students carry out the tasks of elementary school.

## 2. Reading and writing

**Figure 1:**
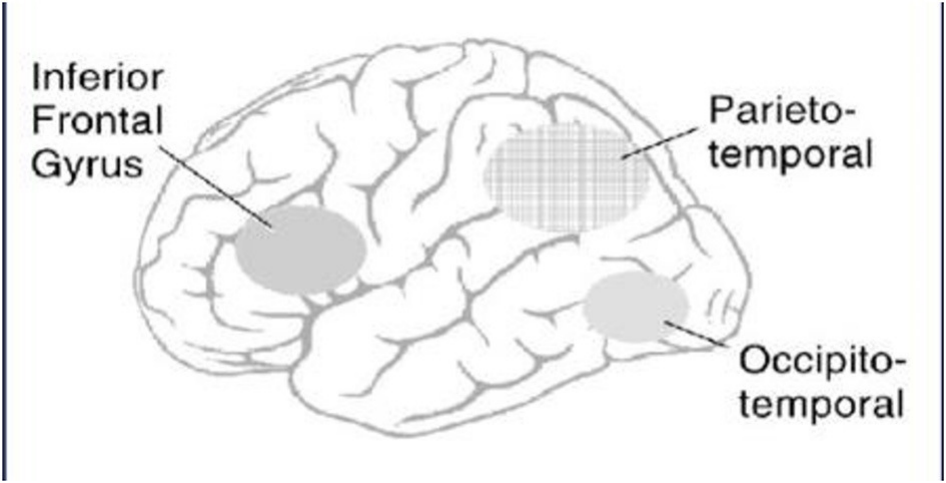
Application of imaging to reading acquisition reveals two posterior brain areas (phonological and visual word form areas), both of which can access word meaning via the ventral frontal lobe. The phonological pathway is related to word sound and the visual word form area is related to the rules for visual words (orthography). The left frontal lobe serves as a means to access semantic information stored in various brain areas.

Reading curricula in elementary school either involve phonics instruction, the whole word method or more recently a “balanced” curriculum that places emphasis on reading for meaning and is based to a large degree on whole words. Brain research has been helpful in resolving the dispute between the phonics and whole word forms of training. In literate adults two different networks connect areas in the posterior part of the brain to attention and brain areas related to meaning (see Figure 1).

One pathway involves obtaining the word name (phonological code) through blending letter sounds into words. For most readers phonics training allows them to handle either unfamiliar or familiar words by blending letter sounds to pronounce them. Words that are familiar from speaking can then be connected to the word meaning. This pathway is based on phonics training and the child's vocabulary from exposure to spoken words (7).

This form of reading is not fluent and does not by itself produce a child likely to read on their own or to enjoy reading (8). The extreme difficulty of reading without a visual word form area is illustrated by adult patients with disconnection of the visual word form area from the primary visual cortex of one hemisphere or both hemispheres. Words presented to the disconnected hemisphere are painfully sounded out letter by letter (9) rather than read fluently (9). Practice in reading induced by assignments and/or reading for pleasure builds the visual word form area. This visual word form area chunks letters into a unit and connects to word meaning (semantics). There is some evidence that the visual word form area is also developed by early reading in the first year of schooling. Thus, phonics reading instruction not only provides a basis for decoding but also helps to develop the visual word form area (10). Reading instruction encroaches on parts of the brain that are initially weakly specialized for tools and close to but distinct from those responsive to faces (10).

The visual word form area may also involve subareas of increasing sensitivity to whole words as one moves from the more early visual with area to later (more anterior) parts of the visual word form area (11). There is also evidence that the development of the visual word form area and its connections to other parts of the brain (connectivity) continues long after decoding has been developed. The time course of connectivity and function of these subareas is under active investigation (12). Exercises that expose children to materials of interest to them help to ensure that they become fluent readers as adults. Individual differences in the reliance on phonology vs. visual word form can be achieved by measuring the accuracy of sounding out nonwords in comparison to the skill of pronouncing exception words, that do not follow the common rules of orthography (13, 14). These measures could be useful to teachers to adapt their methods to ensure that each child reaches reading fluency, but may not be useful in some languages.

Children who are diagnosed as dyslexic, that is who fail to learn to read despite having the apparent ability to do so, show reduced activation of both phonological and visual word form areas (15). As expected, a computerized program to teach phonics improved the ability of the dyslexic children to sound out words and increased activation in phonological brain areas (16). In addition, emphasis on letters by spacing of the visual text can also provide help for some dyslexic children (17).

It is likely that writing may also help to develop the visual word form area. Cursive writing requires the child to develop specific movements for each letter. The emphasis on the letter as a constituent of the word may help develop both the phonological and the teaching visual word form area. In addition, there is some evidence that teach cursive writing may provide better overall memory for the material than would be the case with typing (18). This may support the idea of teaching cursive to foster development of reading skill as well as improved memory.

## 3. Number

The number sense allows animals and human infants to come into the world with a primitive understanding of small numbers. Babies can even perform simple calculation. For example, infants look longer when adding a puppet to the display of one puppet produces a single puppet than when it correctly reveals two puppets (19). Thus babies, like adults, can be puzzled by an error in the visual display of small quantities and this detection activates part of the executive attention network [(20) described in the attention section of this paper].

When adults are asked to determine which of two digits is larger they are faster the larger the distance between the two. We believe this result arises from representation of quantity within the brain's parietal cortex that is called the number line. It is a goal of specific training to develop and expand this representation of quantity. Wilson et al. (21) used a race game which required number comparison. Performance on number comparison did improve, but a subsequent study showed that it did not improve more than a control condition and did not generalize to other math skills (22).

**Figure 2:**
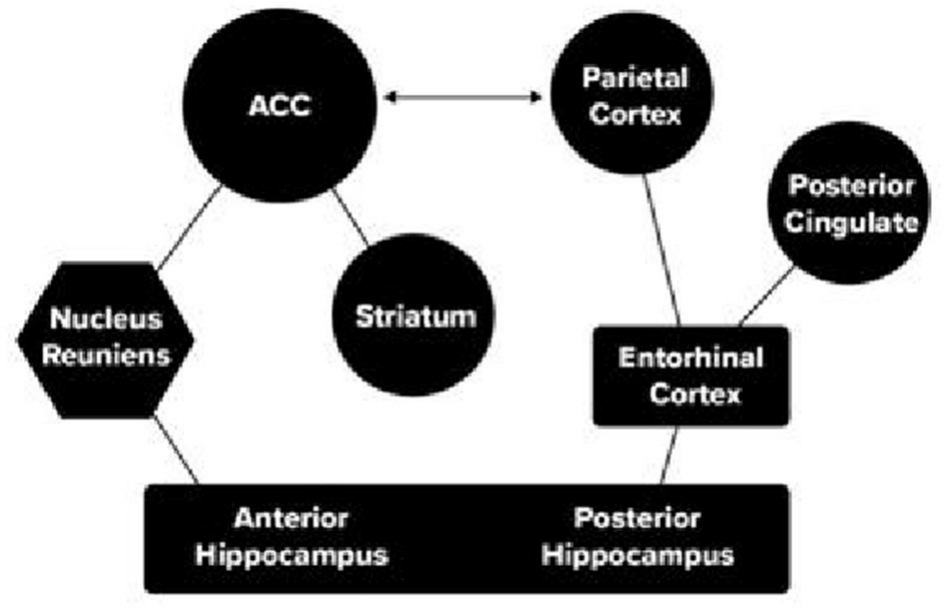
Two pathways between attention nodes (circles) and memory (rectangles), anterior pathway between attention and memory (hexagon). Reprinted with permission from Frontier Neuroscience (24).

A four-week training program using several games, including the race game used in the Wilson et al. study, produced improved appreciation of quantity and strengthened connections in a brain pathway between the parietal lobe and hippocampus (23). This pathway also plays a role in retrieval of newly learned associations (see Figure 2).

Although much research remains it does seem possible to aid in the development of the number line that forms a basic understanding of quantity and serves as a framework for early arithmetic. Another important step for the teacher is to ensure that the concept of quantity is connected to the language network to allow exact calculation (25). This effort could involve discussion of sample problems designed to have the student articulate their concept of quantity in their own language and thus help in the development of the connections between the number line and language that is needed for exact calculation (25).

## 4. Attention and learning

Most of school learning depends on paying attention. Viewing attention as having a single unified function creates confusion in applying it to classroom learning. There is no single brain network of attention, and so far, three mainly different brain networks are related to (1) obtaining and maintaining the alert state, (2) orienting to sensory information, and (3) executive control of voluntary behavior, thoughts and feelings. Each of these functions involves largely separate networks of attention (26, 27).

Both the orienting and the executive attention networks have connections with memory formation and retrieval involving the hippocampus, a central node in consolidating and retrieving long term memories. Figure 2 indicates two somewhat separate pathways between attention and memory networks.

The executive attention network connects the Anterior Cingulate Cortex (ACC) through the thalamus to exert control of the anterior hippocampus during storage of information (28). A posterior route between the parietal orienting network and the hippocampus is largely involved in navigation in rodents and more general retrieval from long-term memory in humans (24).

Two forms of training have been found to improve attention networks. One involves young children and is a 5-day adaptation of training used to send chimpanzees to perform work in space (29). It began by teaching 4–6-year-olds to use a joystick and ended with practice in resolving conflict (30, 31). It has been shown to improve the network underlying self regulation and control of cognition and to allow more behavioral control over delay of gratification. A second method involves training the control of attention through forms of mindfulness meditation. Five days of meditation training improved executive attention (32) and two weeks of training produced a strong 4–8 Hz (theta) rhythm over the frontal cortex even when the person is at rest (33). In mice, 4 weeks of near theta stimulation in the ACC changes connectivity near to the site of stimulation (34).

The ability to train attention in childhood through network training and meditation shows promise as a way of helping children to learn. Attention training was used in central Europe to reduce the gap between high-and-low income families and increase school success (1). Training attention either directly through cognitive exercises or through meditation could be used for the same purpose in the US schools where inequality in pre-school education remains high.

## 5. Training mindset and improving function

There is substantial evidence that the beliefs students have about their brain can greatly influence their behavior (35, 36). For example, those with a growth mindset, who believe that intelligence can be changed by effort, show greater ability to attend and learn new material than those with a fixed mindset, who believe intelligence is fixed and effort has little influence (37). In one study, children with fixed mindset show larger frontal activity to negative feedback but sustained their attention less and learned less than those with a growth mindset, who use negative information to sustain learning. In a national sample of 6,320 American adolescents a one-hour mindset intervention delivered on-line in two sessions not only improved their growth mindset but also improved school achievement for low and medium level students. High level achievers showed reduced variability in their already strong grades (38).

A four-week growth mindset training program was designed to enhance foundational, school related, cognitive skills in 7–10-year-old children (39). The training included a one-on-one tutoring program in number skills and on-line games related to the number sense. In comparison with a non-contact control group the trained children not only improved in growth mindset but the improvement in mindset predicted the improvement in later math skills. Prior to and after training an fMRI was given while children performed a math problem solving (addition) task. The gains in growth mindset were strongly associated with increased activation of the dorsal ACC and to a lesser extent the hippocampus. There were also significant connectivity increases between the ACC and the hippocampus.

Thus, change in mindset from a strong intervention strengthened the pathways between attention and memory during mathematics problem solving. These results show that growth mindset not only provides information on the child's attitude but also serves as a possible proxy for the strength of pathways between attention and memory and provides evidence that intervention can be effective in improving their strength.

## 6. Lessons for elementary teachers

Neuroscience does not dictate the correct curriculum for teaching reading, writing or arithmetic.

What it does do is equip the designers of these curricula and those responsible for their execution with a view of the underlying brain structures and changes that are involved when learning this material. These principles can be associated with assessments that reveal how the affected brain structures are working. These assessments do not require the use of brain scans or other neuroscience methods that are critical for knowledge about the brain.

What might teachers do to achieve the needed background for applying neuroscience findings to their work? Jolles and Jolles (5) argue that four themes in neuroscience are critical for obtaining such knowledge. These are:

Theme 1. The nervous system controls and responds to body functions and directs behavior.

Theme 2. Nervous system structure and function are determined throughout life by genes and environment, including the person's own actions.

Theme 3 The brain is the foundation of the mind.

Theme 4: Research leads to understanding that is essential for development of therapies for nervous system dysfunction and helps improve the circumstances under which people learn.

For more general principles of classroom mangement based on psychological research the reader could turn to https://www.apa.org/ed/schools/teaching-learning.

It may be necessary that every school granting credentials to teachers make sure that at least this level of knowledge is available, and every school board might seek to hire and reward teachers with this relevant knowledge.

## Author contributions

Both authors listed have made a substantial, direct, and intellectual contribution to the work and approved it for publication.
